# Baseline Interleukin-6 and -8 predict response and survival in patients with advanced hepatocellular carcinoma treated with sorafenib monotherapy: an exploratory post hoc analysis of the SORAMIC trial

**DOI:** 10.1007/s00432-021-03627-1

**Published:** 2021-04-14

**Authors:** Osman Öcal, Kerstin Schütte, Juozas Kupčinskas, Egidijus Morkunas, Gabija Jurkeviciute, Enrico N. de Toni, Najib Ben Khaled, Thomas Berg, Peter Malfertheiner, Heinz Josef Klümpen, Christian Sengel, Bristi Basu, Juan W. Valle, Julia Benckert, Antonio Gasbarrini, Daniel Palmer, Ricarda Seidensticker, Moritz Wildgruber, Bruno Sangro, Maciej Pech, Jens Ricke, Max Seidensticker

**Affiliations:** 1 Department of Radiology, University Hospital, Ludwig Maximilian University of Munich, Marchioninistrasse 15, 81377 Munich, Germany; 2 Department of Internal Medicine and Gastroenterology, Niels-Stensen-Kliniken Marienhospital, Osnabrück, Germany; 3 Institute for Digestive Research and Department of Gastroenterology, Medical Academy, Lithuanian University of Health Sciences, Kaunas, Lithuania; 4 Department of Medicine II, University Hospital, LMU Munich, Munich, Germany; 5 Klinik Und Poliklinik Für Gastroenterologie, Sektion Hepatologie, Universitätsklinikum Leipzig, Leipzig, Germany; 6 Department of Medical Oncology, Amsterdam University Medical Centers, University of Amsterdam, Amsterdam, The Netherlands; 7 Radiology Department, Grenoble University Hospital, La Tronche, France; 8 Department of Oncology, University of Cambridge, Cambridge, UK; 9 Division of Cancer Sciences and Department of Medical Oncology, The Christie NHS Foundation Trust, University of Manchester, Manchester, UK; 10 Department of Hepatology and Gastroenterology, Charité-Universitätsmedizin Berlin, Campus Virchow Klinikum, Berlin, Germany; 11 Fondazione Policlinico Universitario Gemelli IRCCS, Universita’ Cattolica del Sacro Cuore, Roma, Italy; 12 Molecular and Clinical Cancer Medicine, University Hospitals and Clatterbridge, University of Liverpool, Liverpool, UK; 13 Liver Unit, Clínica Universidad de Navarra, Pamplona, Spain; 14 Departments of Radiology and Nuclear Medicine, University of Magdeburg, Magdeburg, Germany

**Keywords:** Hepatocellular carcinoma, Sorafenib, Interleukin, Response

## Abstract

**Purpose:**

To explore the potential correlation between baseline interleukin (IL) values and overall survival or objective response in patients with hepatocellular carcinoma (HCC) receiving sorafenib.

**Methods:**

A subset of patients with HCC undergoing sorafenib monotherapy within a prospective multicenter phase II trial (SORAMIC, sorafenib treatment alone vs. combined with Y90 radioembolization) underwent baseline IL-6 and IL-8 assessment before treatment initiation. In this exploratory post hoc analysis, the best cut-off points for baseline IL-6 and IL-8 values predicting overall survival (OS) were evaluated, as well as correlation with the objective response.

**Results:**

Forty-seven patients (43 male) with a median OS of 13.8 months were analyzed. Cut-off values of 8.58 and 57.9 pg/mL most effectively predicted overall survival for IL-6 and IL-8, respectively. Patients with high IL-6 (HR, 4.1 [1.9–8.9], *p* < 0.001) and IL-8 (HR, 2.4 [1.2–4.7], *p* = 0.009) had significantly shorter overall survival than patients with low IL values. Multivariate analysis confirmed IL-6 (HR, 2.99 [1.22–7.3], *p* = 0.017) and IL-8 (HR, 2.19 [1.02–4.7], *p* = 0.044) as independent predictors of OS. Baseline IL-6 and IL-8 with respective cut-off values predicted objective response rates according to mRECIST in a subset of 42 patients with follow-up imaging available (IL-6, 46.6% vs. 19.2%, *p* = 0.007; IL-8, 50.0% vs. 17.4%, *p* = 0.011).

**Conclusion:**

IL-6 and IL-8 baseline values predicted outcomes of sorafenib-treated patients in this well-characterized prospective cohort of the SORAMIC trial. We suggest that the respective cut-off values might serve for validation in larger cohorts, potentially offering guidance for improved patient selection.

**Supplementary Information:**

The online version contains supplementary material available at 10.1007/s00432-021-03627-1.

## Introduction

Hepatocellular carcinoma (HCC) develops mostly on the background of chronic inflammation of the liver (El-Serag 2012). Cytokine signaling, including interleukins (IL), plays an intrinsic role in regulating this inflammatory process, and levels of various cytokines have been shown to be increased in patients with HCC compared to cirrhotic patients (Kakumu et al. 1993; Naugler et al. 2007; Porta et al. 2008; Bergmann et al. 2017).

Sorafenib, a multitarget tyrosine kinase inhibitor, has been shown to improve survival in patients with advanced HCC (Llovet et al. 2008). However, therapy benefit is not uniform for each patient. Several biomarkers have been investigated to predict the efficacy of sorafenib in HCC patients (Llovet et al. 2012). Additionally, after a long time with sorafenib being the only systemic treatment option for HCC, several first- and second-line therapies emerged (Bruix et al. 2017; Abou-Alfa et al. 2018; Kudo et al. 2018), and recently, atezolizumab–bevacizumab combination has been shown to be superior to sorafenib in the first-line setting (Finn et al. 2020). However, despite these advances, sorafenib will undoubtedly continue to be an important treatment option, especially where atezolizumab–bevacizumab is unavailable or contraindicated, and find its new role in the complex HCC treatment algorithm. This situation intensified the need for additional biomarkers of sorafenib benefit. A few preclinical studies have shown that IL-6 and IL-8 are related to sorafenib resistance (Kahraman et al. 2019; Lai et al. 2019; Li et al. 2020). However, a study that evaluated the prognostic role of multiple biomarkers in HCC patients treated with sorafenib showed that baseline IL-8 values failed to detect treatment benefit (Miyahara et al. 2011). On the contrary, another study that investigated IL-6 in an Asian HCC cohort showed that pretreatment IL-6 values with a cut-off of 4.58 pg/mL are correlated with overall survival after sorafenib, with high pretreatment levels associated with a poor prognosis (Shao et al. 2017).

SORAfenib in combination with local MICro-therapy guided by gadolinium-EOB-DTPA-enhanced MRI (SORAMIC, EudraCT 2009-012576-27, NCT01126645) is a prospective, phase II, randomized, controlled study in HCC patients with three study arms. In the palliative arm of the study, HCC patients were randomized to sorafenib treatment either alone or combined with Y90 radioembolization (RE), and the addition of RE treatment failed to improve survival compared to sorafenib monotherapy (Ricke et al. 2019). This exploratory post hoc analysis of the palliative arm of the SORAMIC trial aimed to explore the predictive value of baseline IL-6 and IL-8 in patients with advanced HCC receiving sorafenib monotherapy.

## Materials and methods

### Study population

This post hoc analysis was a substudy of the palliative arm of SORAMIC, a prospective, randomized-controlled phase II trial exploring the additional benefit of RE to sorafenib treatment. We selected a subgroup of patients in the palliation arm receiving sorafenib monotherapy only, to eliminate potential effects of other therapies used within the trial (radioembolization). SORAMIC was conducted in 38 centers in Europe and Turkey. The study protocol was approved by the competent authorities as well as the institutional review board, and all patients gave written informed consent.

The inclusion and exclusion criteria for the SORAMIC trial have been described previously (Ricke et al. 2019). In summary, patients aged 18–85 years with a diagnosis HCC in intermediate stage (Barcelona Clinic Liver Cancer [BCLC] stage B, not eligible for TACE) or advanced stage (BCLC C), adequate liver reserve (Child–Pugh scores A to B7), an Eastern Cooperative Oncology Group performance status (ECOG PS) ≤ 2 were eligible. Patients with extrahepatic disease were recruited as long as the disease was liver-dominant and lungs were not involved. Inclusion into this substudy required the availability of a blood sample before the initiation of sorafenib treatment to measure IL-6 and IL-8 values as part of the translational program of the SORAMIC trial.

**Fig. 1:**
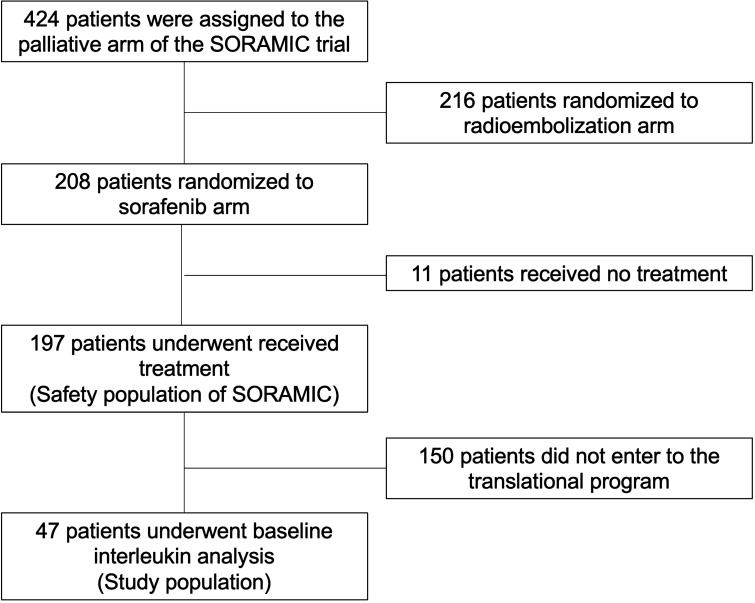
Consort diagram

Of the 208 patients randomized to sorafenib monotherapy in the palliative arm of the SORAMIC trial and 197 patients received sorafenib within the trial. Of these 197 patients, 47 (23.8%) were included in the translational program (study population), and baseline blood samples were available for IL assessment (Fig. 1). There was no significant difference in baseline characteristics between the patients who entered the translational program and the rest of the patients who received sorafenib within the trial (Supplementary Table 1). Baseline characteristics of the study population are listed in Table 1. Forty-one (87.2%) patients had underlying liver cirrhosis. Whereas 23 (48.9%) patients had alcoholic liver disease (two without cirrhosis), 4 (8.5%) had hepatitis B (one without cirrhosis), and 9 (19.1%) had hepatitis C. Thirty-six (76.5%) patients had advanced HCC (BCLC C), and 41 (87.2%) had well-preserved (Child–Pugh A) liver function.

**Table 1:** Patient characteristics

	Number	%
All cohort	47	100
Gender (male)	43	91.4
Race (White)	39	82.9
Liver cirrhosis (yes)	41	87.2
HCC etiology		
Hepatitis B	4	8.5
Hepatitis C	9	19.1
Hepatitis C	9	19.1
Alcohol	23	48.9
ECOG PS		
0	36	76.5
1	11	23.4
Child Pugh score		
A	41 (87.2)	87.2
B	6 (12.7)	12.7
BCLC stage		
B	11 (23.4)	23.4
C	36 (76.5)	76.5

	Median	IQR
Age (years)	66	60.5–72.5
Albumin (g/dL)	38	33–40.7
Total Bilirubin (µmol/L)	15.2	11–21.2
AFP (ng/mL)	108.7	10–1374
IL-6 (pg/mL)	9.7	4.5–17.5
IL-8 (pg/mL)	56.3	34.6–172.9

Patients were randomized in an 11:10 ratio to receive either combination of RE and sorafenib or sorafenib. Patients in the sorafenib arm were started sorafenib treatment after randomization with the starting dose of 200 mg twice daily. If tolerated, the dose was escalated to 400 mg twice daily (target dose) after 1 week. Treatment was continued until tumor progression or the emergence of a drug-related adverse event requiring discontinuation.

Blood samples were obtained before the initiation of the treatment and were stored deep frozen at study core facility and analyzed centrally using Human IL-6 Quantikine ELISA Kit (R&D Sys, Minneapolis, MN, USA; D6050), and Human IL-8/CXCL8 Quantikine ELISA Kit (R&D Sys; D8000C), following the manufacturer’s instructions. Using enzyme-linked immunosorbent assays, serum levels of the IL6 and IL8 were measured.

As a secondary endpoint, in patients with follow-up imaging available for centralized image analysis, follow-up images were evaluated according to modified Response Evaluation Criteria in Solid Tumors (mRECIST) by a board-certified radiologist specialized in gastrointestinal imaging who was blinded to all the clinical information (Llovet and Lencioni 2020).

### Statistical analysis

All statistical analyses were performed using R statistical and computing software, version 3.5.0 (http://www.r-project.org). Categorical variables were reported as counts and percentages, and continuous variables as means and standard deviations. Correlations were evaluated with Chi-square and Fisher’s exact tests, and a *t* test was used to compare two groups. We used the receiver-operating characteristic (ROC) curve to determine the cut-off values for IL-6 and IL-8 that could produce the highest sensitivity and specificity to predict individual survival shorter than the median overall survival. The Kaplan–Meier method was used for estimates of overall survival, and the log-rank test was used to compare survival groups. Cox regression models were used to assess the effects of cofounding factors on overall survival. Variables with a *p* value of < 0.1 in the univariate analyses were analyzed in multivariate Cox regression models to explore prognostic factors of overall survival.

## Results

By the end of the study, 37 (78.7%) patients had deceased, and the median OS in the subset of patients included in this biomarker analysis was 13.8 months.

**Fig. 2:**
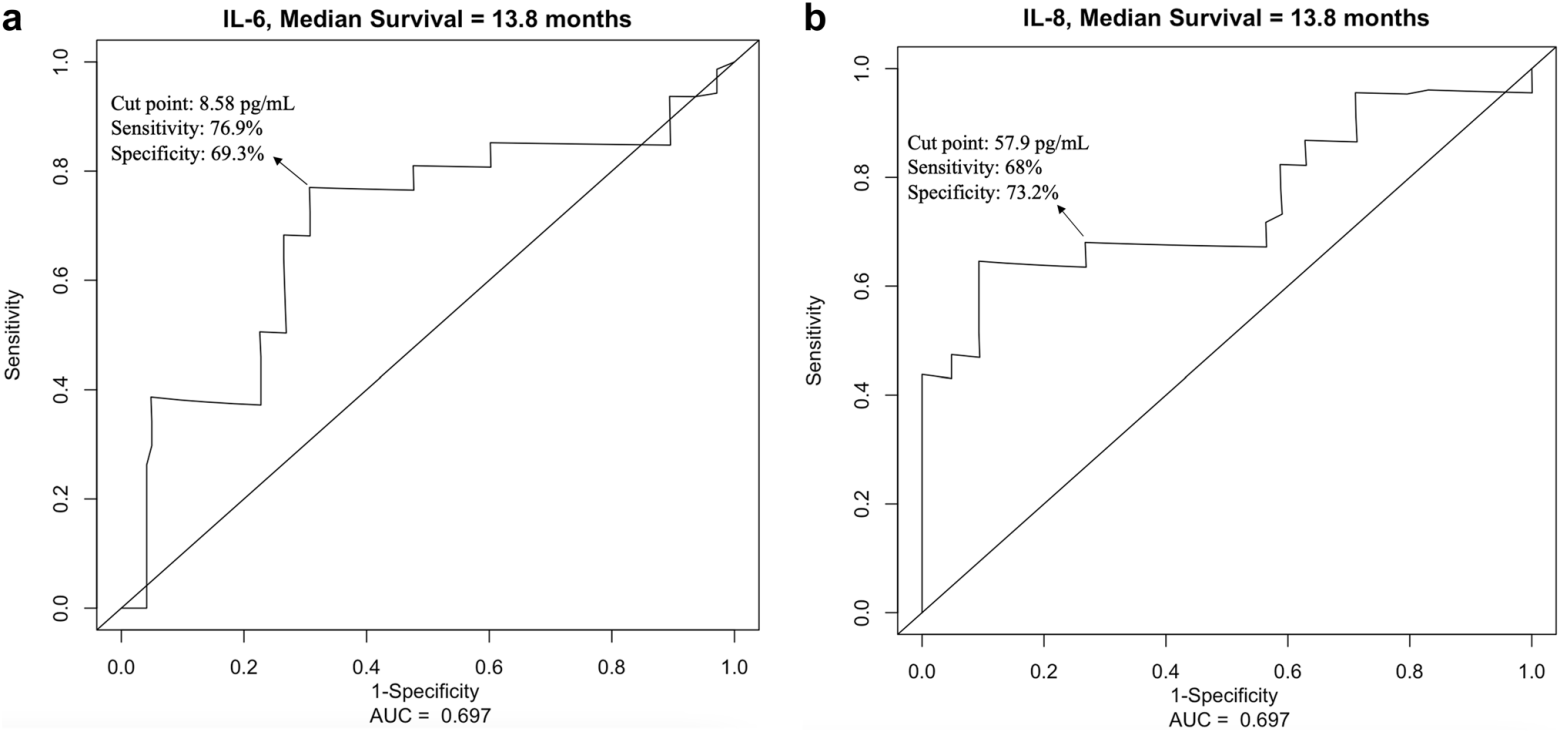
The receiver-operating characteristics (ROC) curve showing the sensitivity and specificity of various cut-off values of baseline: **a** interleukin (IL)-6 and **b** IL-8 levels to analyze the overall survival

Using ROC curve analysis, a cut-off value of 8.58 pg/mL for IL-6 was determined to have the highest sensitivity (76.9%) and specificity (69.3%) to predict survival in these patients (Fig. 2a), whereas an optimal cut-off value of 57.9 pg/mL was defined for IL-8 for a sensitivity of 68% and a specificity of 73.2% (Fig. 2b). Altogether, 26 (55.3%) patients had IL-6 values higher than 8.58 pg/mL, and 23 (48.9%) patients had IL-8 values higher than 57.9 pg/mL. Comparison of baseline characteristics of each subgroup according to IL levels is summarized in Table 2.

**Table 2:** Comparison of baseline characteristics of patients according to IL values

	Overall (*n* = 47)	IL 6 high (*n* = 26)	IL 6 low (*n* = 21)	*p*	IL 8 high (*n* = 23)	IL 8 low (*n* = 24)	*p*
Gender (male)	43 (91.4)	22 (84.6)	21 (100)	0.117	20 (86.9)	23 (95.8)	0.347
Age (≥ 65 years)	28 (59.5)	16 (61.5)	12 (57.1)	0.760	14 (60.8)	14 (58.3)	0.859
Race (White)	39 (82.9)	23 (88.4)	16 (76.1)	0.437	19 (82.6)	20 (83.3)	> 0.99
ECOG PS							
0	36 (76.5)	17 (65.4)	19 (90.5)	0.080	17 (73.9)	19 (79.2)	0.670
1	11 (23.4)	9 (34.6)	2 (9.5)		6 (26.1)	5 (20.8)	
Liver cirrhosis (yes)	41 (87.2)	22 (84.6)	19 (90.5)	0.678	19 (82.6)	22 (91.6)	0.415
HCC etiology							
Hepatitis B	4 (8.5)	3 (11.5)	1 (4.7)	0.617	2 (8.6)	2 (8.3)	> 0.99
Hepatitis C	9 (19.1)	4 (15.3)	5 (23.8)	0.486	6 (26.0)	3 (12.5)	0.286
Alcohol	23 (48.9)	13 (50.0)	10 (43.4)	0.871	11 (47.8)	12 (50.0)	0.881
Previous TACE	15 (31.9)	7 (26.9)	8 (38.0)	0.414	6 (26.1)	9 (37.5)	0.401
Diffuse disease (≥ 10 lesion)	32 (68)	19 (73.0)	13 (61.9)	0.414	17 (73.9)	15 (62.5)	0.401
Median (mean) target lesion size, mm	53 (61.9)	62.5 (71.0)	47 (50.6)	0.084	68 (76.5)	49.5 (47.9)	**0.013**
Portal vein infiltration	28 (59.5)	16 (61.5)	12 (57.1)	0.760	16 (69.5)	12 (50.0)	0.171
Extrahepatic spread	5 (10.6)	3 (11.5)	2 (9.5)	> 0.99	3 (13.0)	2 (8.3)	0.666
Child–Pugh score							
A	41 (87.2)	21 (80.7)	20 (95.2)	0.204	18 (78.3)	23 (95.8)	0.097
B	6 (12.7)	5 (19.2)	1 (4.8)		5 (21.7)	1 (4.2)	
BCLC stage							
B	11 (23.4)	4 (15.3)	7 (33.3)	0.180	4 (17.4)	7 (29.2)	0.493
C	36 (76.5)	22 (84.6)	14 (66.7)		19 (82.6)	17 (70.8)	
Beyond up-to-7 criteria	42 (89.3)	24 (92.3)	18 (85.7)	0.644	21 (91.3)	21 (87.5)	> 0.99
Total bilirubin ≥ 17 µmol/L	15 (31.9)	11 (42.3)	4 (19.0)	0.120	10 (43.4)	5 (20.8)	0.095
Albumin < 36 g/L	16 (34.0)	13 (50.0)	3 (14.2)	**0.013**	10 (43.4)	6 (25.0)	0.181
AFP ≥ 400 ng/mL	17 (36.1)	10 (38.4)	7 (33.3)	0.731	9 (39.1)	8 (33.3)	0.848
Objective response	16 (38.0)	5 (19.2)	11 (46.6)	**0.007**	4 (17.4)	12 (50.0)	**0.011**

Bold type indicates statistical significance

Univariate analysis of clinical and pathological variables conducted by stratifying patients according to these cut-off values showed that high baseline IL-6 was associated with albumin values of < 36 g/L (*p* = 0.013), whereas high baseline IL-8 was associated with larger maximum tumor diameter (*p* = 0.013). In addition, although the difference was not significant, there were more patients with high IL-6 in patients with larger tumors and ECOG 1; and high IL-8 in patients with total bilirubin ≥ 17 µmol/L.

**Fig. 3:**
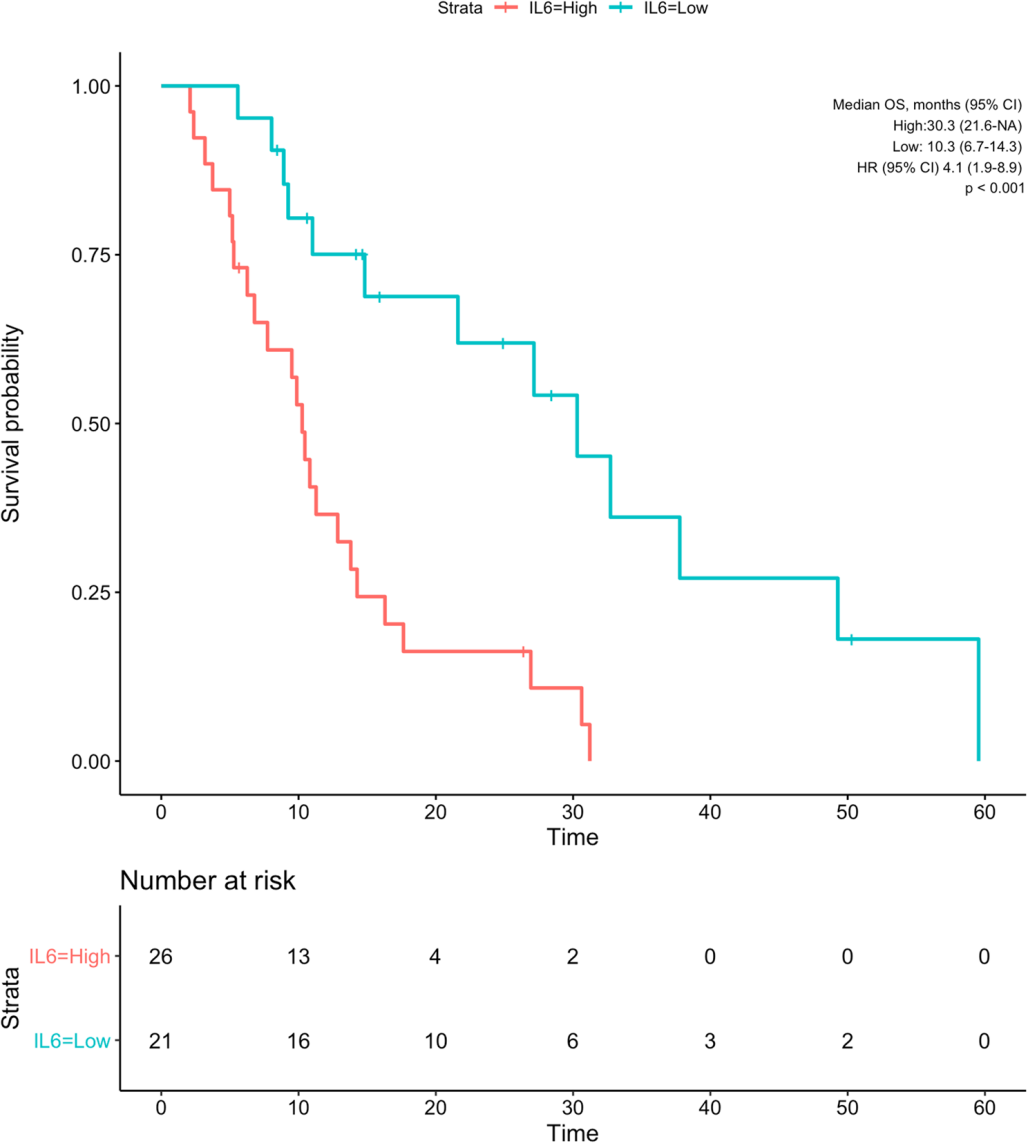
Kaplan–Meier curve showing overall survival of patients grouped by baseline IL-6 values according to cut-off of 8.58 pg/mL

**Fig. 4:**
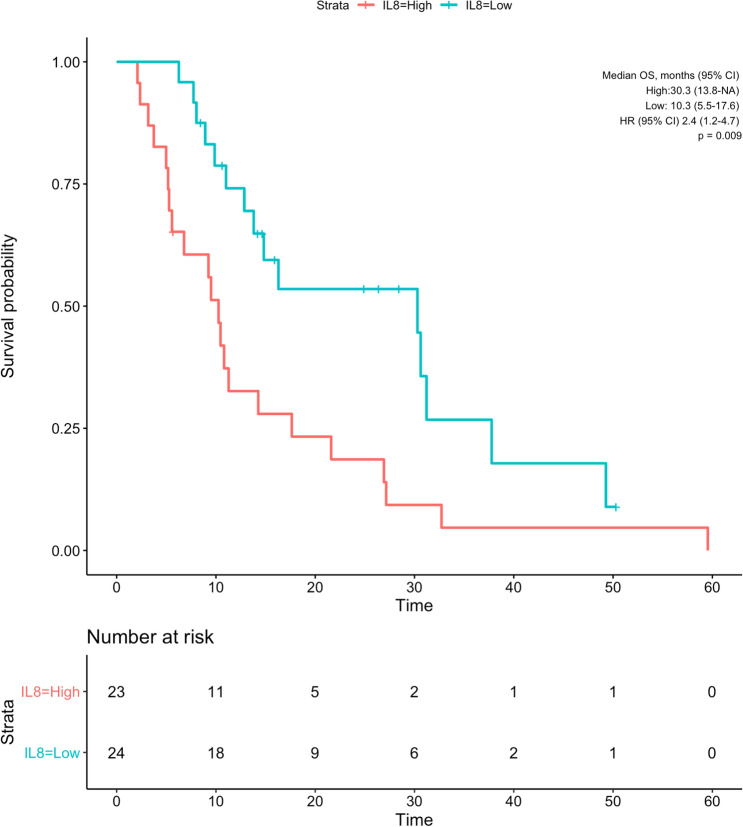
Kaplan–Meier curve showing overall survival of patients grouped by baseline IL-8 values according to cut-off of 59.7 pg/mL

The median overall survival of patients with low IL-6 was 30.3 months (CI 95% 21.6—NA), while patients with high IL-6 had a median overall survival of 10.3 months (95% CI 6.7–14.3; *p* < 0.001; Fig. 3). Similarly, patients with low IL-8 (30.3 [95% CI 13.8—NA] months) had significantly longer overall survival than patients with high IL-8 (10.3 [95% CI 5.5–17.6] months; *p* = 0.009; Fig. 4).

Besides IL-6 and IL-8 values, baseline albumin value ≥ 17 g/L (*p* = 0.008) and tumor diameter ≥ 65 mm (*p* = 0.021) were associated with overall survival, whereas there was a trend for better survival in patients with total bilirubin < 17 g/L (*p* = 0.058) and portal vein invasion (*p* = 0.099). There was no correlation between underlying liver disease and overall survival. Multivariate Cox regression analysis revealed that baseline high IL-6 (HR, 2.99 [95% CI 1.22–7.3]; *p* = 0.017) and high IL-8 (HR, 2.19 [95% CI 1.02–4.7]; *p* = 0.044) values were the only independent predictors of shorter overall survival (Table 3).

**Table 3:** Univariate and multivariate analyses of factors associated with overall survival

Parameter	Univariate analysis		Multivariate analysis	
	HR (95% CI)	*p* value	HR (95% CI)	*p* value
High IL-6	4.1 (1.9–8.9)	** < 0.001**	2.99 (1.22–7.3)	**0.017**
High IL-8	2.4 (1.2–4.7)	**0.009**	2.19 (1.02–4.7)	**0.044**
Sex (male vs. female)	0.79 (0.28–2.3)	0.667		
Age (≥ 65 vs. < 65 years)	1.1 (0.55–2.1)	0.85		
ECOG PS (1 vs. 0)	0.63 (0.28–1.4)	0.263		
Cirrhosis (yes vs. no)	0.63 (0.24–1.7)	0.35		
Hepatitis B etiology (yes vs. no)	1.1 (0.34–3.7)	0.865		
Hepatitis C etiology (yes vs. no)	1.5 (0.68–3.1)	0.335		
Alcohol etiology (yes vs. no)	0.61 (0.3–1.2)	0.164		
TACE history (Yes vs. No)	1.2 (0.61–2.5)	0.578		
PVI (yes vs. no)	0.55 (0.27–1.1)	0.099	0.66 (0.29–1.53)	0.337
Child–Pugh score (B vs. A)	1.5 (0.59–4.1)	0.377		
BCLC stage (C vs. B)	0.57 (0.26–1.2)	0.155		
Beyond up-to-7 (yes vs. no)	1.5 (0.44–4.8)	0.536		
Tumor diameter (≥ 65 vs. < 65 mm)	2.2 (1.1–4.4)	**0.021**	1.31 (0.54–3.18)	0.545
AFP (≥ 400 vs < 400 ng/mL)	1.1 (0.54–2.4)	0.745		
Diffuse disease (≥ 10 lesions)	1.3 (0.64–2.7)	0.457		
Extrahepatic disease	1.6 (0.48–5.5)	0.431		
Albumin	0.37 (0.17–0.77)	**0.008**	0.72 (0.29–1.8)	0.483
Bilirubin	0.51 (0.25–1)	0.058	0.83 (0.38–1.84)	0.649

Bold type indicates statistical significance

The estimated rates of survival at 6 months and 12 months were 95.2% and 66.6%, respectively, in patients with IL-6 < 8.58 pg/mL and 100% and 66.6% in patients with IL-8 < 57.9 pg/mL.

**Fig. 5:**
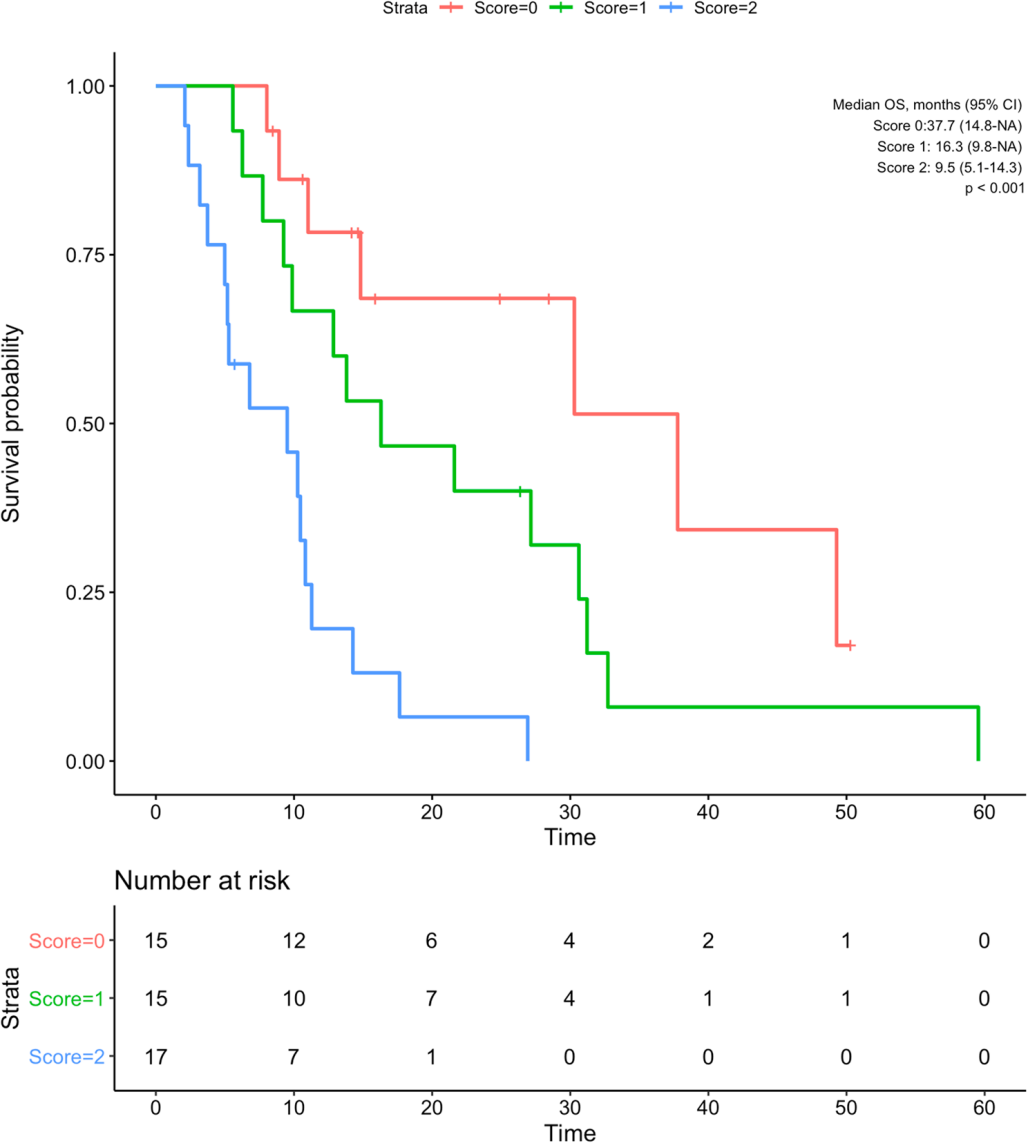
Kaplan–Meier curve showing overall survival of patients according to interleukin score

Patients were scored according to IL levels as follows: both IL-6 and IL-8 lower than cut-off values (score 0), one of IL-6 or IL-8 higher than the cut-off (score 1), and both higher than the cut-off (score 2). Fifteen patients had score 0, 15 had score 1, and 17 had score 2. While the median OS of patients with score 0 was 37.7 (CI 95% 14.8—NA) months, score 1 was 16.3 (CI 95% 9.8—NA) months, and score 2 was 9.5 (CI 95% 5.1–14.3) months (Fig. 5).

For 42 (89.3%) patients, follow-up images were available. Response assessment according to mRECIST revealed an objective response in 16 (38.0%) patients. Patients with IL-6 values lower than cut-off had a significantly higher objective response rate than patients with IL-6 ≥ 8.58 pg/mL (46.6% vs. 19.2%, *p* = 0.007). Similarly, low IL-8 values were also significantly associated with a higher objective response rate (50.0% vs. 17.4%, *p* = 0.011).

## Discussion

In the presented exploratory study, we define baseline levels of IL-6 and IL-8 as prognostic biomarkers of overall survival in patients with advanced HCC undergoing sorafenib treatment by identifying cut-off values of 8.58 pg/mL and 57.9 pg/mL for IL-6 and IL-8, respectively. Baseline IL-6 and IL-8 levels with respective cut-off values remained the only independent predictors of overall survival after adjusting for multiple prognostic factors in the multivariate analysis. In addition, these cut-off values were also associated with objective response in follow-up imaging.

Previous studies have shown that higher IL-6 and IL-8 levels are associated with increased HCC risk in patients with chronic liver disease (Wong et al. 2009; Chien et al. 2011) and correlated with advanced disease stages (Sanmamed et al. 2014; Wang et al. 2016; Sun et al. 2019) and worse liver function in patients with HCC (Chan et al. 2012; Jang et al. 2012). High baseline IL-6 and IL-8 values have been also shown to correlate with treatment response and overall survival in patients who received minimally invasive locoregional therapies (Jang et al. 2012; Carpizo et al. 2014; Seidensticker et al. 2017). A study that investigated the prognostic role of baseline IL-8 under sorafenib treatment in a Japanese HCC cohort, in which 86.7% of the patients had viral hepatitis, showed no correlation between baseline IL-8 levels and treatment response or survival (Miyahara et al. 2011). A single study in the literature explored IL-6 as a predictor in HCC patients receiving sorafenib (55 and 73 patients in exploration and validation cohorts) showed a cut-off value of 4.28 pg/mL could predict survival (HR, 2.5 [1.3–5.0], *p *= 0.005) in an Asian cohort (Shao et al. 2017). Most patients (98.1%) in this study had viral hepatitis. The application of this cut-off value to our cohort failed to detect a survival benefit (data not shown). ROC analysis of the SORAMIC cohort revealed the cut-off value of 8.58 pg/mL for IL-6 with a sensitivity of 76.9% and specificity of 69.3% to predict individual survival longer than the median survival of the cohort. This discrepancy may represent the differences between Asian and Western cohorts and the need for different cut-off values for each. For example, while the most common underlying etiology was the alcoholic liver disease with 48.9% of the patients, and the rate of viral hepatitis was 27.6% in our cohort; the vast majority of patients (86.7–98%) in previously mentioned Asian cohorts had viral hepatitis (Miyahara et al. 2011; Shao et al. 2017). However, testing for the influence of etiology of underlying disease in our analysis did not identify a significant difference in IL values. This might be the result of small numbers and a combination of different causative factors. The cut-off value described in our study is in the range of previously reported cut-off values for IL-6 to detect survival benefit of HCC patients who underwent transarterial chemoembolization (10 pg/mL) and radioembolization (6.53 pg/mL) (Jang et al. 2012; Seidensticker et al. 2017). Furthermore, the identified cut-off values for IL-6 and IL-8 were also correlated with objective response during follow-up (according to mRECIST), demonstrating the capacity of baseline IL-6 and IL-8 as potential prognostic biomarkers.

IL-6 is a pro-inflammatory cytokine and induces the production of acute-phase reactants in the liver. It is also associated with cell proliferation, resistance to apoptosis and chemotherapeutics, and metastasis (Naugler et al. 2007; Schmidt-Arras and Rose-John 2016). IL-8 is a macrophage-derived cytokine that induces tumor angiogenesis and recruitment of immunosuppressive cells to the tumor (Koch et al. 1992; Fousek et al. 2020). Preclinical studies have shown that IL-6/STAT3 signaling contributes to sorafenib resistance in HCC cell lines, and blockage of IL-6 increases cytotoxicity of sorafenib (Niu et al. 2018; Lai et al. 2019; Li et al. 2020). Similarly, inhibition of IL-8 signaling reduces stem cell population in HCC and increases sorafenib sensitivity of tumor cells (Kahraman et al. 2019). Although both IL-6 and IL-8 are related to sorafenib resistance, mechanisms of action are through different pathways. The difference in the mechanisms of IL-6 and IL-8 was partially represented by our cohort. Whereas IL-6 was associated with albumin values and had a tendency to be higher in patients with worse performance status and larger tumors, IL-8 was associated with tumor diameter and partly correlated with bilirubin values. These findings are consistent with the previous studies (Jang et al. 2012; Sanmamed et al. 2014). In addition to these, when patients were scored according to IL levels (2 being both high, 1 either IL-6 or IL-8 high, and 0 both low), those with higher scores had significantly shorter overall survival. This highlights the importance of recognizing both baseline IL-6 and IL-8 values as interacting prognostic factors to cover both of liver inflammation/injury and tumor-related factors.

Recently, Imbrave150 trial has shown that the combination of atezolizumab with bevacizumab improved both overall and progression-free survival as compared to sorafenib (Finn et al. 2020). Despite the promising results of this and similar studies, not all patients are ideal candidates for atezolizumab and bevacizumab therapy. For example, patients with autoimmune diseases or organ transplant recipients were excluded from the Imbrave150 trial, as well as patients with Child–Pugh class B liver function. The high cost, iv. application need, and toxicity of the immunotherapies might prevent rapid worldwide implementation of these therapies. Additionally, atezolizumab–bevacizumab has shown to be not cost-effective as compared to sorafenib (Wang et al. 2021), which will restrict its use, especially in resource-limited countries. Furthermore, sorafenib will remain an important second-line treatment option in patients who progressed after atezolizumab–bevacizumab (Kudo 2021), and optimized treatment decision-making needs to be supported. For example, the survival rate at 12 months of atezolizumab–bevacizumab-treated patients was 67.2% in the IMbrave150 study (Finn et al. 2020), and patients with IL-6 or IL-8 values lower than cut-off values had similar rates of overall survival (66.6%) at the same time point in our study under sorafenib. For better utilization of limited resources, baseline IL values can potentially serve in risk stratification and patient allocation into therapies once validated. Therefore, further evaluation of the additional benefit of therapies suppressing IL pathways to current therapies in HCC patients with high baseline IL values is warranted. Besides this, baseline measurements of IL-6 and IL-8 should be used to stratify patients between treatment arms in future phase 3 trials for new drugs to improve patient selection for the therapy and avoid confounders.

This study has some limitations. Blood sampling for the translational program was not mandatory in the SORAMIC trial, and 18 of 38 centers participated in the translational study, and samples for IL analyses were available in 23.8% of the patients received sorafenib monotherapy. However, our study represents the single cohort proving the prognostic role of baseline IL-6 and IL-8 values after sorafenib treatment of HCC in a Western cohort comprising high-quality data collected prospectively within a multicenter randomized trial. Nevertheless, further validation of these cut-off values in a larger cohort of patients receiving sorafenib treatment clearly is needed.

In conclusion, our study identified the prognostic value of baseline IL-6 and IL-8 values in advanced HCC patients receiving sorafenib treatment. The described cut-off values might be useful for individual patient allocation between different therapies in the era of checkpoint inhibitors or aggressive combination treatments. Additionally, baseline cytokine measurements should be included in future trials the assessing benefit of a new therapeutic regimen in advanced HCC. However, further validation of these cut-off values in larger cohorts is warranted.

## Supplementary Information

Below is the link to the electronic supplementary material.

Supplementary file1 (DOCX 18 kb)

## Data Availability

Data are available through corresponding author upon reasonable request.
