# Supplementary material for: Baseline Interleukin-6 and -8 predict response and survival in patients with advanced hepatocellular carcinoma treated with sorafenib monotherapy: an exploratory post hoc analysis of the SORAMIC trial
Source: J Cancer Res Clin Oncol. 2021 Apr 14;148(2):475–85. doi: 10.1007/s00432-021-03627-1 (PMC8800931; doi:10.1007/s00432-021-03627-1)
Supplement: Supplementary file 1 — Supplementary file1 (DOCX 18 kb) [file 432_2021_3627_MOESM1_ESM.docx]

**Supplementary table 1.**

|  | **Patients within the translational program**  **(n=47)** | **Rest of sorafenib received patients within SORAMIC (n=150)** | **p value** |
| --- | --- | --- | --- |
| Gender (Male) | 43 (91.4) | 128 (85.3) | 0.400 |
| Age (≥65 years) | 28 (59.5) | 85 (56.6) | 0.725 |
| ECOG PS   - 0 - ≥1 - Missing | 36 (76.5)  11 (23.4)  - | 97 (64.6)  52 (34.7)  1 (6.7) | 0.141 |
| Liver cirrhosis (yes) | 41 (87.2) | 115 (76.7) | 0.119 |
| HCC etiology   - Hepatitis B - Hepatitis C - Alcohol | 4 (8.5)  9 (19.1)  23 (48.9) | 18 (12.0)  35 (23.3)  60 (40.0) | 0.683  0.547  0.279 |
| Portal vein infiltration | 28 (59.5) | 67 (44.6) | 0.074 |
| Extrahepatic spread | 5 (10.6) | 32 (21.3) | 0.133 |
| Child Pugh score   - A - B | 41 (87.2)  6 (12.7) | 139 (92.7)  11 (7.3) | 0.247 |
| BCLC stage   - A - B - C - Missing | -  11 (23.4)  36 (76.5)  - | 3 (2.0)  44 (27.3)  102 (68.0)  1 (6.7) | 0.286* |
| Total bilirubin ≥17 µmol/L | 15 (31.9) | 61 (40.7) | 0.282 |
| Albumin < 36 g/L | 16 (34.0) | 44 (29.3) | 0.540 |
| AFP ≥ 400 ng/mL | 17 (36.1) | 28 (18.7) | 0.686 |

* C vs. A&B
